# Life Expectancy in Pleural and Peritoneal Mesothelioma

**DOI:** 10.1155/2017/2782590

**Published:** 2017-01-23

**Authors:** Robert Shavelle, Kate Vavra-Musser, Jessica Lee, Jordan Brooks

**Affiliations:** Life Expectancy Project, San Francisco, CA, USA

## Abstract

*Background*. Mesothelioma is a rare cancer with a historically dire prognosis. We sought to calculate life expectancies for patients with pleural or peritoneal mesothelioma, both at time of diagnosis and several years later, and to examine whether survival has improved in recent years.* Methods*. Data on 10,258 pleural and 1,229 peritoneal patients from the SEER US national cancer database, 1973–2011, were analyzed using the Cox proportional hazards regression model.* Results*. The major factors related to survival were age, sex, stage, grade, histology, and treatment. Survival improved only modestly over the study period: 0.5% per year for pleural and 2% for peritoneal.* Conclusions*. Life expectancies were markedly reduced from normal, even amongst 5-year survivors with the most favorable characteristics and treatment options.

## 1. Introduction

Mesothelioma is a rare cancer of the mesothelial cells, accounting for fewer than 1% of all cancers [1, 2]. Mesothelial cells make up the mesothelium, a membrane which forms the lining of body cavities including the thoracic cavity (pleura), abdominal cavity (peritoneum), and heart sac (pericardium) or forms a membranous cover for the internal male reproductive organs (tunica vaginalis of testis).

Pleural mesothelioma is the most common form (80–85% of cases) and often presents with shortness of breath, chest pain, or fatigue. Peritoneal mesothelioma (10–15% of cases) can affect the organs in the abdomen, with late symptoms including abdominal swelling, nausea, vomiting, and bowel obstruction. The other two sites make up less than 5% of cases.

Most cases are due to asbestos exposure [3, 4], though the correlation has been found to be stronger in pleural than in peritoneal cases [5, 6]. The risk of development is related to the extent and length of exposure. People exposed to asbestos at an early age, for a long period of time, and at higher levels are more likely to develop the cancer. Malignancy develops slowly, and the latency period (time between first exposure and diagnosis) is usually 20 to 50 years. Unfortunately, the risk of developing mesothelioma does not decrease upon cessation of exposure.

About 3,000 new cases are diagnosed each year in the United States [2]. It is much more common in men than in women (due to occupational exposure such as construction [7]) and in Caucasian or Hispanic races than in African American or Asian [2]. Due in part to the long latency period, mesothelioma is rarely diagnosed in persons under age 45 (4% of cases), and about two-thirds of patients are age 65 or older. The incidence rate for new cancers increased from the 1970s to the early 1990s but has since stabilized and decreased slightly. The decrease has been more pronounced in men than women and is thought to be related to changes in workplace exposure to asbestos. In some other countries the rate is still increasing.

There are three main histologic types:

1. Epithelioid (histologic code 9052) composes roughly 60% of cases. Tumors of this cell type tend to be easier to identify and also easier to remove with surgery, and thus persons with this type tend to have a better survival prognosis [8, 9].
2. Fibrous sarcomatoid (9051), roughly 25% of cases, is more aggressive than epithelial, and patients often do not respond as well to treatment.
3. Biphasic/mixed (9053), roughly 15% of cases, have both epithelial and fibrous sarcomatoid cells and thus can be more difficult to treat than the epithelioid variety [10].

Other (rare) histologies include desmoplastic, lymphohistiocytoid, deciduoid, anaplastic, multicystic, and well-differentiated papillary mesothelioma [11, 12].

As with most cancers, mesothelioma has historically been staged as localized, regional, or distant metastasis (LRD staging system). More recently a specialized TNM system has been used [13], with associated summary staging (I through IV) based on the individual values of T, N, and M. Of the cases in the US National SEER database used here for which staging information exists (roughly 80% of cases), LRD was the primary staging system through 2003 for pleural and 2009 for peritoneal, after which times the TNM system became almost exclusively used. Two clinical systems specifically designed for staging mesothelioma, the Butchart [14] and Brigham [15] systems, are not represented in the SEER database and were therefore not used in our analysis. Because our interests here were on life expectancy (i.e., long-term survival) and changes in survival over time, we opted to use the staging system (LRD) covering the longest period of the available data even though it is less useful clinically, especially in peritoneal cases.

The stage (extent) of a mesothelioma is known to be an important factor in determining treatment options. But other factors, such as whether the cancer is resectable, and a person's general health and treatment preferences, also play a role. Mesothelioma is difficult to treat, regardless of whether the cancer is resectable. In general, most stage I and some stages II and III pleural mesotheliomas are potentially resectable, but there are exceptions. Whether a tumor is resectable is also based on the subtype, where it is located, how far it has grown into nearby tissues, and whether the person is healthy enough to undergo surgery. Some clinical views on resection have been referred to as “controversial” [16], and of course treatment is constantly evolving based on recent research [17].

Prior studies on the factors related to survival in pleural mesothelioma have identified: age, sex, grade, stage, histology, surgery, radiotherapy, diagnosis year, and race [18, 19]. Other characteristics, including genomic factors such as BAP1 mutations [20], have been suggested as related to survival but are beyond the scope of the SEER national database used here. For peritoneal mesothelioma, the factors include age [21], sex [21, 22], stage [23], histology [21, 23], surgery [22–24], chemotherapy [23, 24], and whether the patient was diagnosed in a hospital with a thoracic surgery unit [21].

Previous research has identified factors related to survival and reported on various survival probabilities, including the median survival time, but has not provided life expectancies (the average survival times). As explained by Stephen Jay Gould, who himself had abdominal mesothelioma, the median does not capture the effect of outliers, nor does it provide the prognosis for someone who has already survived the first (high-risk) year after diagnosis [25].

It is common in cancer research to report 5-year survival figures or medians but not to provide life expectancies. The latter require survival times or probabilities for the lifespan and thus are not as readily available or estimated. In mesothelioma the prognosis is generally poor, and thus complete follow-up information on survival is more easily obtained. Life expectancies can therefore be calculated.

For both pleural and peritoneal mesotheliomas in the SEER (US SEER National) Cancer Database, we calculated life expectancy based on various patient characteristics. We did so both from the time of initial diagnosis and also conditioned upon patient survival to 2 or 5 years after diagnosis (i.e., in those who had survived the period with the highest mortality risk). We also investigated whether survival has improved in recent years.

## 2. Materials and Methods

The Surveillance, Epidemiology, and End Results (SEER) database [26], managed and maintained by the National Cancer Institute (NCI), is the largest source of information on cancer incidence and survival in the United States. The 2013 SEER submission contains data on approximately 8.2 million cancer cases diagnosed between 1973 and 2011. The registries that provide patient information for SEER represent approximately 28% of the US population (based on the 2010 census). Data is collected on primary tumor site, morphology, stage, treatment, follow-up, and patient demographics, among other things. The SEER program is the only comprehensive source of population-based cancer information in the United States that also includes stage of cancer at time of diagnosis.

NCI maintains and updates both the SEER database and the SEER*∗*stat software application, a specialized statistical program designed for use with the database. We used SEER*∗*stat version 8.1.5 (released March 31, 2014), which includes patient diagnoses in 1973 to 2011 and mortality follow-up through 2011.

There were 15,917 cases of mesothelioma as defined by histologic ICD-O-3 codes 9050–9059. All but 3 of the cases were histologic codes 9051 (fibrous mesothelioma), 9052 (epithelioid mesothelioma), 9053 (biphasic mesothelioma), or 9050 (mesothelioma not otherwise specified [NOS]). We exported the data from SEER*∗*Stat in order to use more advanced statistical software for our analyses.

We restricted attention to the 13,410 pleural and 1,634 peritoneal cases. The former were defined as cases with primary site in the pleura (site C38.4), while the latter as those with primary site in the peritoneum, retroperitoneum, and overlapping lesions of peritoneum and retroperitoneum (sites C48.0–C48.2, C48.8). We then selected only patients with (1) positive, microscopically confirmed histology, (2) known follow-up time, and (3) age 40 or older. The last condition was invoked in order to concentrate on the bulk of the data, avoid unusual cases of patients diagnosed at young ages, and thus improve the modelling of the effect of advancing age on survival. The final dataset contained 11,487 cases: 10,258 pleural (89%) and 1,229 peritoneal (11%).

For simplicity and consistency over time, we used the SEER historic (or summary) staging system: localized, regional, and distant metastasis (LRD). The TNM staging system was introduced to SEER only in 2004. We converted AJCC (6th and 7th editions) staging values to LRD as follows: stage I was considered to be localized; II, III, and IV (M0) regional; and IV (M1) distant.

In SEER, the specific grading systems used are not listed, and in mesothelioma cases there was a high amount of missing data for grade (90%). We therefore excluded grade from the final multivariate models. Information as to whether the patient was treated by chemotherapy (either systemic or local-regional) is not presently given in SEER. This is a significant limitation; indeed, in one recent series more than half of patients with mesothelioma were treated with systemic chemotherapy [16].

We analyzed the survival data using the Kaplan-Meier survival estimator and univariate and multivariate Cox proportional hazard regression models [27]. Analyses were completed using SAS software version 9.4 [28]. Variables of interest included sex (binary; male, female), age (continuous; year), race (binary; Caucasian, other races), treatment (categorical; radiation only, surgery only, both radiation and surgery, neither radiation nor surgery), grade (categorical: grades 1–4), LRD stage (categorical: localized, regional, distant), and histology (categorical: fibrous, epithelial, biphasic, mesothelioma not otherwise specified). All variables were first assessed independently in univariate models, separately by primary site (pleural and peritoneal). We then fit multivariate models.

The Cox model allows for estimation of the survival function for any given combination of values of the variable in the models (i.e., levels of the covariates). That is, one can construct a customized survival curve for any given patient characteristics. We considered various representative groups. For each group, life expectancy was calculated as the area under the survival curve [29], which is equivalent to constructing a life table [30]. In the instances where the curve did not reach 0%, we conservatively imputed thereafter a constant (rather than increasing) mortality rate in older age. This choice was largely immaterial, affecting the computed values by at most 0.2 years. Life expectancies were calculated at three time points: at diagnosis and also at 2 and 5 years after diagnosis. For the latter two time points, we used the same Cox model as for time 0 (at diagnosis) but recalibrated conditional on survival to 2 or 5 years. Life expectancy was compared with that of the age- and sex-specific US general population [30].

## 3. Results

Patient characteristics are shown in Table 1. In keeping with prior research, we found that 81% of pleural cases were male; in contrast, only 56% of the peritoneal cases were male. More than half the pleural cases were in patients over age 70, compared with 29% of peritoneal cases. Most cases were in Caucasian patients (92%) and were of unspecified histology (56% of pleural and 66% of peritoneal).

Table 2 compares the empirical (Kaplan-Meier) and modelled (Cox) survival probabilities. For example, in males aged 50–79 with localized pleural mesothelioma, the empirical results show that 46% survived one year from diagnosis, 22% survived 2 years from diagnosis, and 7% survived 5 years from diagnosis. The analogous percentages from the simple Cox model are similar: 45%, 25%, and 9%. These figures demonstrate the high early mortality (100% − 46% = 54% died in the first year). They also demonstrate that mortality persists thereafter. Notably, of the 46% who survive the first year, only 22%/46% = 48% survive the second year; also, of the 22% who survive 2 years, only 7%/22% = 32% survive to 5 years. That is, even amongst persons who survive 1 or 2 years after diagnosis (i.e., conditional survival), the future prognosis remains poor. We return to this point in the context of conditional life expectancy, when we calculate life expectancy for persons who have survived 2 or 5 years from diagnosis.

As can be seen, the empirical and modelled survival percentages are very close in most instances (excepting perhaps the few instances with very small sample sizes), indicating that even a crude Cox model (one with terms only for age, sex, and stage) may be a fair predictor of overall survival. This similarity lends confidence to the more involved Cox models considered later. In general, a Cox model with additional covariates will produce more accurate survival figures.

Univariate models and the final multivariate Cox models are shown in Table 3, separately for pleural and peritoneal cases. In the pleural case, the hazard ratio (HR) for males was 1.28, indicating that, all else being equal, males had 28% higher mortality than females. Also, Caucasians had 3% lower risk ( = 100% − 97%) compared with other races. Overall, the mortality risk was shown to increase at a rate of 2% per year of age (i.e., someone aged 61, for example, had 2% higher mortality than an otherwise similar person aged 60). Regarding stage, persons with distant metastases had 38% higher risk compared with persons whose cancer was localized, and those with regional metastases had 36% higher risk. Persons with the fibrous type had 58% higher risk than those with the epithelial type, and persons with biphasic type had 43% higher risk. In terms of treatment, those who had radiation therapy alone had 22% higher risk than those who did not require either radiation or surgery.

We chose to include several statistically and practically insignificant factors (e.g., race in the pleural model, with HR = 0.97 and *p* value 0.45) in order to document that their effects were modest or negligible and to allow for comparison between pleural and peritoneal cases. As noted earlier, grade was missing in approximately 90% of cases and thus was not included in the final multivariate models. Consistent with expectations, the hazard ratios for missing values (for variables stage, histology, grade, and therapy) were intermediate to those for levels with known values indicating that the missing category represents a blend of the known categories. For example, with respect to histology, patients with NOS tumors (not otherwise specified) had 22% higher risk (HR = 1.22), between the 1.00 for epithelial and 1.43/1.58 for biphasic/fibrous. Overall, patients with the epithelioid histology, all else being equal, had the most favorable outcome, while those with fibrous sarcomatoid histology had the worst (1.58 or 2.57).

Remarkable in the final models is the modest effect of stage. For example, in pleural cases, distant stage (metastasis) had only 1.38 times the mortality risk of localized stage. Perhaps equally remarkable is the very small effect of diagnosis year. Mortality rates over the past 40 years have fallen only 0.5% per year ( = 100% − 99.5%) in pleural cases and roughly 2% ( = 100% − 98%) per year in peritoneal cases. Of clinical importance are the noted effects of treatment. Patients who received both radiation and surgery had much smaller risk than those who received neither. Pleural patients had 70% (0.70) of the risk compared with those who had no radiation or surgery, or a reduction of 30%, while peritoneal patients had 58% of the risk, a reduction of 42%. It should be noted, however, that treatment assignment was not randomized, indicating that these results cannot be generalized to provide treatment recommendations. We return to this issue in the discussion.

Table 4 shows life expectancies stratified by time since diagnosis, age, sex, and stage. All life expectancies in Table 4 are for Caucasian patients with epithelial mesothelioma, diagnosed in 2010, and treated with both radiation and surgery. Consider a male aged 40, recently diagnosed with distant pleural mesothelioma. His life expectancy from the time of diagnosis is approximately 4 additional years, rather than the 39 years that would be obtained in the general population. If this male survives to 2 years after diagnosis, his life expectancy at that time would be 8 additional years, much higher than the initial 4 years but still much lower than the comparable GP figure (37 years). His conditional life expectancy improved markedly (from 4 to 8) because he survived a very high-risk period (the first 2 years after diagnosis). If he survives to 5 years after diagnosis, his life expectancy at that time would be 11 additional years (compared with 34 years in the GP). The other scenarios of the Table show the same trend. Namely, life expectancy in mesothelioma is much reduced from normal, even amongst persons who survive the first 2 or 5 years after diagnosis. It is of course possible to calculate life expectancies for any other combinations of variable levels from the models shown in Table 3.

The computed life expectancies summarize the very poor survival prospects for mesothelioma patients. Even for persons with the most favorable characteristics displayed here (age 40, localized cancer, amenable to treatment with radiation, and surgery), the life expectancy at time of diagnosis is only 11 years for males and 15 for females. Unlike most other cancers, survival does not markedly improve for those who have survived the first several years following their diagnosis.

## 4. Discussion

As expected, age, sex, and stage were major factors associated with survival in both pleural and peritoneal mesothelioma. In addition, and consistent with the prior literature [8, 9], we found that persons with the epithelioid histology, all else being equal, had better survival, and those with fibrous sarcomatoid histology had worse survival [10].

As indicated in the results, patients in the SEER database were not randomized to treatment. Treatment was decided on a case-by-case basis, as determined by the treating physicians, the patients themselves, and other factors. Therefore, the fact that patients who received both radiation and surgery had much smaller risk (70% or 58%, resp.) does not indicate that all patients should receive both treatments. Instead, it is possible that only the patients who were sufficiently healthy qualified for and received both surgery and radiation. Treatment may therefore serve more as an indicator of health than an independent variable that can be modified. Further investigation on this topic may warrant the construction of a propensity score to reflect the likelihood of receiving a particular treatment, then including this score in the final multivariate model. In the present study, including a propensity score would have rendered our models less useful in describing any particular case, where treatment is known with certainty.

As noted, the SEER database does not currently provide information as to whether the patient was treated with any form of chemotherapy. This is a significant limitation, as such therapy is now known to be increasingly advantageous, especially in special cases or in concert with other therapies [31–34]. The results given here are therefore not specific to this mode of treatment.

Under the assumption of proportional hazards, the Cox model (using all data) gives estimates that are more precise than that of the (small) cohort approach of Kaplan-Meier. That is, under model assumptions, the standard errors of the estimates are smaller. Also, importantly, under the Cox model one can calculate survival figures for various combinations of risk factors that would otherwise result in very small (Kaplan-Meier) cohorts with large standard errors or perhaps even combinations not present in the existing data. For example, one could consider white males, aged 45, diagnosed in 2009, fibrous histology, peritoneal, localized tumor, and treated only with radiation.

Relatively simple main effects multivariate models on the entire datasets were fitted here. More complicated models are possible, including those both using a subset of the data, perhaps also based on time since diagnosis, and including various interaction terms. The model fitting process allows for the adjustment for many risk factors and omnibus testing of their possible effects. For example, as reported here, we documented and tested for a secular trend in survival (0.5% and 2% per year, resp.) while simultaneously accounting for possible calendar-year differences in patient age, sex, race, histology, stage, and treatment modality.

A significant limitation of the SEER data is that the main information is collected only at the time of diagnoses and initial treatments. Remission and relapse status at future time points are not known. The results given here are unbiased at time of diagnosis, but only unbiased at later time points (e.g., at 2 and 5 years after diagnosis) if the patient is “average” with respect to the extant survivors. That is, the calculations here cannot be made specific to a known remission status at later time points. A further significant limitation, as discussed, is that information is not kept in SEER on whether the patient had chemotherapy. Another limitation is that the LRD staging system used here does not reflect current clinical practice and in addition may be less useful for peritoneal cancers. A final one is that data on comorbid factors (e.g., diabetes, heart disease) is not included. Again, therefore, the results are applicable to an “average” patient.

## Figures and Tables

**Table 1:** Demographics. All figures are column percentages unless noted otherwise.

Variable	Category	Pleural	Peritoneal
Sample size (*n*)		10,258	1,229
Deaths (percentage)		9,418 (92%)	989 (83%)

Diagnosis year	1973–1989	17	24
	1990–1994	9	8
	1995–1999	11	10
	2000–2004	25	22
	2005–2009	27	27
	2010-2011	11	10

Age (years)	40–49	4	13
	50–59	13	24
	60–69	27	27
	70–79	36	28
	80+	20	1

Sex	Male	81	56
	Female	19	44

Race	White	92	92
	Other	8	8

Stage	Localized	16	5
	Regional	26	10
	Distant	37	41
	Missing	21	44

Histology	NOS/other	56	66
	Fibrous (9051)	10	2
	Epithelial (9052)	29	29
	Biphasic (9053)	6	3

Grade	1 or 2	3	9
	3 or 4	8	7
	Missing	89	83

Radiation	No	82	95
	Yes	16	4
	Missing	2	1

Cancer-directed surgery	No	70	55
	Yes	26	42
	Missing	3	3

Therapy combined	No radiation or surgery	61	52
	Radiation only	9	3
	Surgery only	20	40
	Radiation and surgery	7	3
	Missing	3	3

**Table 2:** Comparison of empirical (Kaplan-Meier) and modeled (Cox) survival percentages. Sample sizes (*n*) refer to the Kaplan-Meier cohorts. The sample sizes for these two Cox models were *n* = 10,258 cases for pleural (79% staged) and *n* = 1,229 cases for peritoneal (56% staged). Note: the two Cox models used for this table contained terms only for age, sex, and localized/regional/distant/missing.

	Survival percentage					
Method	Empirical			Modeled		
Time since diagnosis (years)	1	2	5	1	2	5
Pleural, all	40	18	5	n/a	n/a	n/a
Localized (*n* = 1,638)	41	19	6	n/a	n/a	n/a
Regional (*n* = 2,632)	40	17	4	n/a	n/a	n/a
Distant (*n* = 3,848)	32	12	3	n/a	n/a	n/a
Males aged 30–49						
Localized (*n* = 42)	58	43	34	63	45	25
Regional (*n* = 68)	53	23	10	60	40	21
Distant (*n* = 123)	42	21	6	54	34	15
Males aged 50–79						
Localized (*n* = 979)	46	22	7	45	25	9
Regional (*n* = 1,688)	43	19	4	41	21	7
Distant (*n* = 2,413)	35	14	3	34	15	4

Peritoneal, all	50	35	18	n/a	n/a	n/a
Localized (*n* = 61)	74	53	26	n/a	n/a	n/a
Regional (*n* = 122)	55	41	19	n/a	n/a	n/a
Distant (*n* = 508)	40	26	11	n/a	n/a	n/a
Males aged 30–49						
Localized (*n* = 5)	80	60	40	69	39	23
Regional (*n* = 5)	60	40	0	54	39	0
Distant (*n* = 22)	42	37	21	48	43	21
Males aged 50–79						
Localized (*n* = 31)	68	50	18	65	46	18
Regional (*n* = 55)	56	42	15	57	40	16
Distant (*n* = 238)	30	19	9	31	19	9

**Table 3:** Hazard ratios (*p* values) for variables in the univariate and multivariate Cox proportional hazards regression survival models for pleural and peritoneal mesothelioma.

Variable	Categories	Pleural		Peritoneal	
		Univariate	Multivariate	Univariate	Multivariate
Sex	Male	1.324 (<0.001)	1.28 (<0.001)	1.50 (<0.001)	1.38 (0.001)
	Female	1 (ref)	1 (ref)	1 (ref)	1 (ref)

Race	White	1.00 (0.92)	0.97 (0.45)	0.76 (0.026)	0.78 (0.044)
	All other races	1 (ref)	1 (ref)	1 (ref)	1 (ref)

Age (years)	(Continuous)	1.02 (<0.001)	1.02 (<0.001)	1.03 (<0.001)	1.02 (<0.001)

Stage	Localized	1 (ref)	1 (ref)	1 (ref)	1 (ref)
	Regional	1.10 (0.003)	1.36 (<0.0001)	1.37 (0.073)	1.49 (0.026)
	Distant	1.33 (<0.001)	1.38 (<0.001)	1.92 (<0.001)	2.04 (<0.001)
	Missing	1.24 (<0.001)	1.25 (<0.001)	1.352 (0.046)	1.73 (0.001)

Histology	Fibrous (9051)	1.56 (<0.001)	1.58 (<0.001)	2.02 (0.002)	2.17 (0.001)
	Biphasic (9053)	1.44 (<0.001)	1.43 (<0.001)	1.23 (0.25)	1.444 (0.046)
	NOS (9050)	1.33 (<0.001)	1.22 (<0.001)	1.18 (0.021)	1.10 (0.227)
	Epithelial (9052)	1 (ref)	1 (ref)	1 (ref)	1 (ref)

Grade	1 or 2	1 (ref)	Not included	1 (ref)	Not included
	3 or 4	2.21 (<0.001)		4.28 (<0.001)	
	Missing	1.59 (<0.001)		2.77 (<0.001)	

Diagnosis year	(Continuous)	1.00 (0.001)	0.995 (<0.001)	0.98 (<0.001)	0.98 (<0.001)

Therapy	No radiation or surgery	1 (ref)	1 (ref)	1 (ref)	1 (ref)
	Radiation only	1.22 (<0.001)	1.22 (<0.001)	1.32 (0.168)	1.15 (0.507)
	Surgery only	0.67 (<0.001)	0.70 (<0.001)	0.54 (<0.001)	0.62 (<0.001)
	Radiation and surgery	0.61 (<0.001)	0.70 (<0.001)	0.54 (0.003)	0.58 (0.010)
	Missing	0.84 (0.003)	0.85 (0.007)	0.78 (0.159)	0.80 (0.220)

**Table 4:** Life expectancy in mesothelioma by time since diagnosis (TSD, in years), location (pleural or peritoneal), age, sex, and stage (L = localized, R = regional, and D = distant). The other covariates in the final multivariate model (see Table 3) were set to race = white, histologic type = epithelial, diagnosed in 2010, and treatment by radiation and surgery. Life expectancies for other combinations of the covariates can similarly be computed. GP = general population life expectancies, 2010.

Location	Age	Male				Female			
		GP	L	R	D	GP	L	R	D
TSD = 0 years									
Pleural	40	39	6	5	4	42	9	7	6
	50	30	5	4	3	33	7	6	5
	60	22	3	3	2	25	5	4	3
	70	14	2	2	2	17	4	3	2
	80	8	2	1	1	10	3	2	2

Peritoneal	40	39	11	8	6	42	15	11	8
	50	30	9	6	4	33	12	9	6
	60	22	7	5	3	25	9	7	5
	70	14	5	3	2	17	7	5	3
	80	8	4	2	2	10	5	4	2

TSD = 2 years									
Pleural	42	37	11	9	8	41	13	12	10
	52	28	9	8	7	31	11	10	9
	62	20	7	6	6	23	9	8	7
	72	13	6	5	5	15	8	7	6
	82	7	5	4	4	9	6	5	5

Peritoneal	42	37	13	10	8	41	17	13	10
	52	28	11	9	7	31	14	11	9
	62	20	9	7	6	23	12	9	7
	72	13	8	6	4	15	10	8	6
	82	7	6	5	4	9	8	6	5

TSD = 5 years									
Pleural	45	34	13	12	11	38	16	14	13
	55	25	12	10	9	29	14	13	11
	65	18	10	9	8	20	12	11	10
	75	11	9	8	7	13	10	9	8
	85	6	6	6	6	7	9	8	7

Peritoneal	45	34	13	10	8	38	16	13	10
	55	25	10	8	7	29	13	11	8
	65	18	9	7	6	20	11	9	7
	75	11	7	6	5	13	9	8	6
	85	6	6	5	4	7	7	6	5

## Additional Points

Mesothelioma is a rare cancer often with dire prognosis. The present work examined the factors related to survival and found that survival has not improved in recent decades. In addition, life expectancies, rather than merely median survival times, were reported, including those for patients who have already survived 2 or 5 years after diagnosis. Contrary to common wisdom in many cancers, even long-term survivors do not have a normal life expectancy.
